# Synthetic cannabinoid for the treatment of severe chronic noncancer pain in children and adolescents

**DOI:** 10.1080/24740527.2022.2132138

**Published:** 2022-11-28

**Authors:** Naiyi Sun, Natasha Cunha, Shawnee Amar, Stephen Brown

**Affiliations:** a Department of Anesthesia and Pain Medicine, The Hospital for Sick Children, Toronto, Ontario, Canada; b Department of Anesthesiology and Pain Medicine, University of Toronto, Toronto, Ontario, Canada

**Keywords:** cannabinoid, chronic pain, children

## Abstract

**Background:**

The prevalence of chronic pain in children and adolescents is high. In some patients, it can be severe and refractory to conventional treatment options. There is increasing interest in the use of cannabinoids for therapeutic purposes in children and adolescents. Nabilone, a synthetic cannabinoid, is approved in Canada for the treatment of nausea and vomiting associated with chemotherapy. It can also be used off label for treatment of chronic pain.

**Aims:**

This study aims to characterize the use of nabilone for severe chronic pain in a pediatric population.

**Methods:**

This is a retrospective cohort study of patients 18 years or younger who were prescribed nabilone for chronic pain in a tertiary multidisciplinary pediatric chronic pain clinic between July 1, 2013, and June 30, 2017.

**Results:**

During the 4-year study period, we screened the charts of 507 patients and identified a total of 28 patients (5.5%) who were treated with nabilone as part of their chronic pain treatment. Common indications for nabilone treatment include mixed neuropathic/nociceptive pain, abdominal pain, neuropathic pain, and spasticity. In all patients, nabilone was prescribed as an adjunctive treatment. Seven patients (25%) reported a slight improvement in pain symptoms. Side effects were reported by 21.4% of patients. The most common reported side effects were sedation and cognitive slowing.

**Conclusions:**

Adjunctive treatment with nabilone may improve pain symptoms in a subset of pediatric chronic pain patients. Further research investigating the long-term safety and efficacy of nabilone in the treatment of chronic pain in children is needed.

## RÉSUMÉ

**Contexte**: La prévalence de la douleur chronique chez les enfants et les adolescents est élevée. Chez certains patients, elle peut être sévère et réfractaire aux options de traitement conventionnelles. On s'intéresse de plus en plus à l'utilisation de cannabinoïdes à des fins thérapeutiques chez les enfants et les adolescents. Nabilone, un cannabinoïde synthétique, est approuvé au Canada pour le traitement des nausées et des vomissements associés à la chimiothérapie. Il peut également être utilisé hors étiquette pour le traitement de la douleur chronique.**Buts** : Cette étude vise à caractériser l'utilisation du Nabilone pour les douleurs chroniques sévères chez une population pédiatrique.**Méthodes** : Il s'agit d'une étude de cohorte rétrospective de patients âgés de 18 ans ou moins à qui du Nabilone a été prescrit pour la douleur chronique dans une clinique pédiatrique multidisciplinaire tertiaire de la douleur chronique entre le 1er juillet 2013 et le 30 juin 2017.**Résultats** : Au cours de la période d'étude de quatre ans, nous avons examiné les dossiers de 507 patients et répertorié un total de 28 patients (5,5%) traités par Nabilone dans le cadre de leur traitement de la douleur chronique. Les indications courantes pour le traitement par Nabilone comprennent les douleurs neuropathiques/nociceptives mixtes, les douleurs abdominales, les douleurs neuropathiques et la spasticité. Chez tous les patients, Nabilone a été prescrit comme adjuvant. Sept patients (25 %) ont rapporté une légère amélioration des symptômes de la douleur. Des effets secondaires ont été rapportés par 21,4 % des patients. Les effets indésirables les plus fréquemment rapportés étaient la sédation et le ralentissement cognitif.**Conclusions** : Le traitement adjuvant par Nabilone peut améliorer les symptômes de la douleur chez un sous-ensemble de patients pédiatriques souffrant de douleur chronique. D’autres études sur l'innocuité et l'efficacité à long terme du Nabilone dans le traitement de la douleur chronique chez les enfants sont nécessaires.

## Introduction

Chronic pain is highly prevalent among children and adolescents, affecting approximately one in five children.^1^ Chronic pain can occur in children due to underlying health conditions (e.g., sickle cell disease, rheumatological conditions, genetic syndromes) or as a primary pain condition (primary headache, complex regional pain syndrome). It can lead to debilitating effects beyond pain, impacting many aspects of daily life, including sleep, emotions, school attendance, social interactions, and physical activities. The total cost of chronic pain in adolescents is estimated to be $19.5 billion annually in the United States.^2^ The optimal treatment model for chronic pain in children involves a multidisciplinary approach involving pharmacological, physical, and psychological strategies. However, severe chronic pain in children can be challenging to treat despite these treatment strategies. Response to current pharmacological treatment strategies is highly variable. There is very little literature available examining the efficacy and outcomes of pharmacological interventions in young people with chronic pain.^3^ In adults, only one out of every three patients who receive treatment for neuropathic pain experiences an analgesic effect, defined as ≥30% pain reduction.^4,5^ Alternative treatment strategies are needed for patients who are refractory to conventional treatment options.

Cannabinoids are biologically active constituents of cannabis or synthetic compounds with an affinity for or activity at the cannabinoid receptors. There is evidence in adults that cannabinoids can be helpful in the treatment of human immunodeficiency virus neuropathy, diabetic neuropathy, posttraumatic or postsurgical neuropathic pain, and mixed central and peripheral neuropathic pain states.^6^ Cannabinoids are also currently recommended as a third-line treatment option for chronic neuropathic pain in the 2014 revised Canadian Pain Society neuropathic pain guidelines.^7^

Nabilone is an orally administered synthetic cannabinoid and analogue of delta-9-tetrahydrocannabinol (THC), which is the main psychoactive component in cannabis. It is licensed in Canada and United States as an antiemetic for management of nausea and vomiting associated with cancer chemotherapy. The use of nabilone for treatment of chronic pain is off-label. Nabilone acts as an agonist of two endogenous cannabinoid receptors, CB1 and CB2. Stimulation of the CB1 receptor, which is located in neuron terminals throughout the nervous system, reduces neuronal excitability. Stimulation of the CB2 receptor results in anti-inflammatory effects. Nabilone has also been shown to improve sleep in adults likely due to the sedative effects of THC.^8^

The 2018 legalization of medical marijuana in Canada and 18 U.S. states has led to a gap between increased interest in this treatment modality by patients and families and limited evidence for its use in the pediatric population. A 2017 systematic review to identify the evidence base of cannabinoids as a medical treatment in children and adolescents showed that evidence for benefit was strongest for chemotherapy-induced nausea and vomiting, with increasing evidence of benefit for epilepsy. However, there is insufficient evidence for its use in chronic pain conditions such as spasticity and neuropathic pain.^9^ Cannabinoids were found to be well tolerated in a systematic review of randomized trials of cannabinoids for treatment of chronic noncancer pain in adults. Adverse effects were generally mild to moderate in severity.^10^ Current evidence on the use of medical cannabinoids for pain in children is limited to only one case report in children with complex regional pain syndrome (CRPS) and a small trial in children with complex motor disorders.^11,12^

There is a paucity of literature available to guide clinicians on the use of medical cannabinoids in children and adolescents. The American Academy of Pediatrics currently opposes dispensing medical cannabis to children and adolescents; however, it does recognize that it may be an option for children with severely debilitating conditions and for whom current therapies are inadequate.^13^ In this study, we present a retrospective chart review of a single-center clinical experience on the use of nabilone for a small series of pediatric patients with severe chronic noncancer pain over a 4-year period.

## Methods

The study design was a retrospective cohort study, analyzing data on all patients aged ≤18 years followed in our chronic pain clinic who were prescribed nabilone between July 1, 2013, and June 30, 2017, at a single tertiary pediatric teaching hospital (The Hospital for Sick Children, Ontario, Canada). This study was approved by the institutional research ethics board (No. 1000059383) and consents were waived given the deidentified and retrospective nature of the data collected.

The pediatric chronic pain clinic at The Hospital for Sick Children provides interdisciplinary pain management to children and adolescents. Patients were identified through a search of all new patients seen in clinic during this time period through the clinic database. Patients were selected for review if they were (1) prescribed nabilone for pain treatment and (2) ≤18 years old at the time of prescription. Patients prescribed nabilone for indications other than chronic pain (e.g., antiemetic) were excluded from the study.

Data were collected from hospital electronic medical records, including clinic notes, letters, and scanned prescriptions. For each patient, we collected data including patient’s age, sex, weight, primary pain diagnosis, location of pain, and any concurrent medical diagnosis. Information on nabilone prescriptions included dose prescribed and duration of treatment. Nabilone’s effect on pain and other symptoms, reported side effects, and reasons for discontinuation were collected from patient and family reports that were documented in the clinic notes. We also collected information on other pain medications used including other forms of prescribed cannabinoids such as cannabidiol (CBD) oil and nonpharmacological treatments provided. All comments related to nabilone treatment in the chart were captured. The chronic pain clinic includes a total of four physicians who all prescribed nabilone as part of their practice. There was no formal treatment protocol established for nabilone in the chronic pain clinic. It was introduced as an adjunctive treatment for patients at the discretion of the prescribing pain physician. All data were extracted from charts and entered into an anonymized database. For this study, the initial visit is considered the chronic pain clinic visit where nabilone was prescribed. If nabilone was prescribed between clinic visits, the chronic pain clinic visit immediately prior to the prescription was used as the initial visit.

Descriptive statistics are presented. Numerical variables are displayed as median and interquartile range (IQR) and categorical variables are presented as numbers and percentages.

## Results

During the 4-year study period, we screened charts from 507 patients and identified a total of 28 patients (5.5%) who were treated with nabilone as part of their chronic pain treatment. The median age of our patient cohort at the start of nabilone treatment was 14.0 years (IQR:11.8–16) and 75% of patients were female (Table 1).

**Table 1.** Patient demographics and baseline characteristics.

Characteristic	Category	*N* = 28
Age at initiation of cannabinoids (years), median (IQR)		14.0 (11.8–16)
Age (years), *n*/total *N* (%)	6–9	3/28 (10.7)
	10–12	7/28 (25.0)
	13–15	5/28 (17.9)
	16–18	13/18 (46.4)
Sex, *n*/total *N* (%)	Male	7/28 (25.0)
	Female	21/28 (75)
Weight at initiation of cannabinoids (kg), median (IQR)		51.0 (31.9–60.5)
Maximal nabilone daily dose (mg), median (IQR)		1.5 (0.9–2.0)
Associated diagnosis, *n*/total *N* (%)	Ehlers-Danlos syndrome	4/28 (14.3)
	Cerebral palsy	3/28 (10.7)
	Intestinal dysmotility disorder	3/28 (7.1)
	Postural orthostatic tachycardia syndrome	3/28 (7.1)
	Systemic lupus erythematosus	1/28 (3.6)
	Chronic recurrent multifocal osteomyelitis	1/28 (3.6)
	Chronic pancreatitis	1/28 (3.6)
	Lesch-Nyhan syndrome	1/28 (3.6)
	Seizure disorder	1/28 (3.6)
	Thoracic outlet syndrome	1/28 (3.6)
	Ichthyosiform erythroderma	1/28 (3.6)
	Netherton’s syndrome	1/28 (3.6)
Concurrent mental health diagnosis, *n*/total *N* (%)	Anxiety	10/28 (35.7)
	Depression	6/28 (21.4)
	Posttraumatic stress disorder	1/28 (3.6)
Primary pain location, *n*/total *N* (%)	Generalized	12/28 (42.9)
	Abdomen	6/28 (21.4)
	Lower extremity	4/28 (14.3)
	Head	3/28 (10.7)
	Upper extremity	2/28 (7.1)
	Back	1/28 (3.7)
Pain diagnosis, *n*/total *N* (%)	Mixed neuropathic/nociceptive pain	9/28 (32.1)
	Abdominal pain	6/28 (21.4)
	Neuropathic pain	5/28 (17.9)
	Spasticity/dystonia	4/28 (14.3)
	Procedural pain	2/28 (7.1)
	Headache	1/28 (3.6)
	CRPS	1/28 (3.6)

IQR = Interquartile range.

The most common primary pain diagnosis associated with nabilone treatment was mixed neuropathic/nociceptive pain (32.1%), followed by abdominal pain (21.4%), neuropathic pain (17.9%), and spasticity (14.3%). The pain location was most commonly described as generalized (42.9%), followed by abdominal (21.4%) and lower extremity (14.3%). The patients frequently had coexisting mental health disorders, including anxiety (35.7%) and depression (21.4%).

Patients were concurrently taking between one and five other pain medications while on nabilone. The number of patients taking one, two, three, and four or more concomitant medications was seven, eight, five, and eight patients, respectively. The most common concurrent pharmacological treatments include anti-epileptics, nonsteroidal anti-inflammatory drugs, opioids, and antidepressants (Table 2). In one patient, nabilone was started to facilitate rapid opioid weaning. All patients had attempted multiple previous pharmacological treatments (between two and five medications) prior to starting nabilone.

**Table 2.** Pharmacological and nonpharmacological treatments used concurrently with nabilone, *N* = 28 patients.

Pharmacological and nonpharmacological treatments	Number of patients, *n* (%)
Opioids	
Hydromorphone	6 (21.4)
Oxycodone	3 (10.7)
Morphine	2 (7.1)
Anti-epileptics	
Gabapentin	6 (21.4)
Pregabalin	6 (21.4)
Antidepressants	
Amitriptyline	3 (10.7)
Nortriptyline	1 (3.6)
Venlafaxine	3 (10.7)
Duloxetine	2 (7.1)
Sertraline	1 (3.6)
Trazadone	1 (3.6)
Nonsteroidal anti-inflammatory drugs	
Celecoxib	6 (21.4)
Ibuprofen	3 (10.7)
Diclofenac	3 (10.7)
Acetaminophen	9 (32.1)
Clonidine	4 (14.3)
Muscle relaxants	
Baclofen	1 (3.6)
Methocarbamol	1 (3.6)
Medical cannabis (CBD oil)	2 (7.1)
Interventions	2 (7.1)
Physiotherapy treatments	10 (35.7)
Psychological treatments	16 (57.1)
Transcutaneous electrical nerve stimulation	2 (7.1)
Acupuncture	1 (3.6)
Hypnosis	1 (3.6)
Massage therapy	1 (3.6)

CBD = Cannabidiol.

The duration of treatment varied widely, from less than one week to more than 12 months in 13 patients. The longest duration of treatment was 29 months. None of the patients started nabilone treatment less than 3 months from the time of chart review. Side effects and effectiveness were evaluated at follow-up visits, which varied between 1 and 6 months after initiation of therapy. Patients had between one and ten follow-up visits prior to terminal of therapy or discharge from care. Nabilone dosing ranged from 0.25 mg once daily up to 2 mg twice daily. The median daily nabilone dose prescribed was 1.5 mg. Due to limited documentation of follow-up pain scores, only 13 patients had documented pain scores both at the start of treatment and at follow-up visits. Seven patients showed a small reduction of between 0.5 and 1.5 on the Numeric Rating Scale in pain scores. Three patients had pain scores that were unchanged. Three patients reported increased pain scores during treatment. Side effects were documented in 6 of the 28 patients. Side effects reported include sedation, cognitive slowing, agitation, nightmares, and dry mouth (Table 3).

**Table 3.** Positive effects, negative side effects, and reason for discontinuing medication documented for patients treated with nabilone.

		Number of patients, *n*/total *N* (%)
Positive effects	Improved pain	7/28 (25.0)
	Improved sleep	7/28 (25.0)
	Improved nausea	3/28 (10.7)
Negative effects	Sedation	3/28 (10.7)
	Cognitive slowing	2/28 (7.1)
	Agitation	2/28 (7.1)
	Nightmare	1/28 (3.6)
	Dry mouth	1/28 (3.6)
Reasons for discontinuation	Not helpful	4/15 (26.7)
	Initially helpful, then no longer helpful	2/15 (13.3)
	Side effects	2/15 (26.7)
	Lack of availability	1/15 (6.7)
	Discontinued by other services	1/15 (6.7)
	Not reported	5/15 (33.3)

## Discussion

The objective of this study was to describe the use of nabilone in a small cohort of children and adolescents with severe chronic pain that was refractory to other treatment strategies. In this retrospective analysis of nabilone prescribing practice over a 4-year period in a pediatric multidisciplinary chronic pain clinic, we found that 5.5% of patients were prescribed a trial of nabilone. In our cohort, there was a slight improvement in pain in a small subset of patients treated with nabilone. It was tolerated without side effects in a majority of the patients, with 21.4% of patients reporting side effects. The most common reported side effects were sedation and cognitive slowing.

Several clinical trials have shown inconclusive results on the use of selective cannabinoid products containing THC for the treatment of chronic neuropathic pain in adults.^14–16^ In other studies, treatment with THC-containing cannabinoids decreased pain in patients with rheumatoid arthritis and neuropathic radiculopathy.^17,18^ The THC-induced analgesia was correlated with a reduction in functional connectivity between the anterior cingular cortex and sensorimotor cortex in functional magnetic resonance imaging.^18^ A Canadian Agency for Drug and Technologies in Health rapid review specifically evaluating the use of nabilone for management of chronic pain found limited evidence that nabilone may be better than placebo or medications such as amitriptyline in relieving chronic pain.^19^

Information on the use of synthetic cannabinoids for chronic pain in children and adolescents is currently limited to only case report and one small randomized trial. Rudich et al. reported the use of dronabinol in two adolescents with neuropathic pain and comorbid major depressive disorder. Dronabinol reduced the affective component of pain by 40% and improved psychosocial functioning after 4 months, although there was a gradual dissipation of effectiveness after 6 months that led to discontinuation.^11^ A second small randomized trial examined the use of CBD enriched oil extract with two different CBD-to-THC ratios on children with complex motor disorders. Participants from both groups reported a statistically significant improvement in pain (Table 4).^12^ Our study adds to the limited available information on the use of nabilone for chronic pain in children and adolescents.

**Table 4.** Summary of previous studies on the use of cannabinoids for chronic noncancer pain treatment in children and adolescents.

Study (country)	Study Design	Patient Characteristics	Intervention	Pain Outcomes
Current study (Canada)	Retrospective cohort study	28 children aged 4 to 16 with chronic noncancer pain	Nabilone 0.25–4 mg/day for variable duration	25% of patients reported a small reduction of between 0.5 and 1.5 on the Numeric Rating Scale in pain scores
Libzon et al.^12^ (Israel)	Randomized noncontrolled trial (no placebo control group)	25 Children aged 1 to 17 years with complex motor disorders	Patients were randomized to two different CBD-enriched 5% oil formulations (CBD-to-THC ratio 6:1 vs. CBD-to-THC ratio 20:1) administered for 5 months	1.41-point reduction in pain out of 10 on visual analog scale in both treatment groups
Rudich et al.^11^ (Canada)	Case report	Two adolescents with CRPS type 1	Dronabinol 5–25 mg/day for 4 months	One participant reported 45% improvement in pain intensity, whereas the second reported no improvement; 50% reduction in affective component of their pain

The use of medical cannabis has become increasingly popular. Studies have shown that 10% to 15% of adult patients with chronic pain use cannabis to improve pain, sleep, and mood.^20^ The scarce data available concerning the pediatric population require clinicians to extrapolate data from adult studies. A simplified Canadian guideline for prescribing medical cannabinoids in primary care recommends the use of pharmaceutically developed products such as nabilone or nabiximols as initial agents because dosing is more consistent.^21^ They suggested that clinicians could consider medical cannabinoids for refractory neuropathic pain if patients have had a reasonable therapeutic trial of at least three prescribed analgesics and have persistent problematic pain despite optimized analgesic therapy. They should be given as an adjunct to other prescribed analgesics. The findings of our study were largely in accordance with these recommendations. The patients in this series had all trialed multiple medications and still experienced pain that was poorly managed by conventional multidisciplinary treatment options prior to starting nabilone. Nabilone was prescribed as an adjunct to other pharmacologic and nonpharmacologic treatments in all patients.

The decision to trial cannabinoids for chronic pain in children and adolescents needs to be carefully balanced with the potential risk of cannabinoids, in particular their risk to the developing brain. The CB1 receptors are particularly concentrated in brain regions critical for executive functioning, reward processing, and memory.^22^ There is evidence from recreational cannabis users suggesting that the chronic use of cannabis may have adverse effects on the developing brain. Neuroimaging studies show that individuals who began using recreational cannabis regularly in adolescence tend to have differences in cortical and subcortical volumes, white matter integrity, and functional connectivity compared to nonusers.^23^ There is also epidemiological evidence to support the view that regular or heavy cannabis use among adolescents and young adults may increase the risk of developing psychotic disorders.^24^ However, there are significant differences between recreational use and medical use of synthetic cannabinoids use, including frequency, potency, route of administration, and number of active ingredients. The lack of research limits our understanding of the long-term effects of medical cannabinoids in children and adolescents.

Currently, there is limited evidence from randomized controlled trials to inform the practice of treating children and adolescents with chronic pain using cannabinoids to improve pain intensity and disability. The potential benefit of cannabinoids in pediatric populations must be balanced with the associated risks. The Canadian Pediatric Society recommends that the use of cannabis for medical purposes in children be evaluated on a case-by-case basis and always with a comprehensive discussion of potential benefits, risks, and goals of treatments.^25^

Without a pediatric guideline that provides specific recommendations on the use of cannabinoids for children and adolescents with chronic pain, we suggest the following steps be considered when prescribing cannabinoids for chronic pain:

1. A comprehensive biopsychosocial pain assessment needs to be performed, including a psychological assessment for history of psychotic disorder, anxiety, and substance abuse and family history of psychotic disorders.
2. When considering cannabinoids, physicians must engage patients and families in an informed consent discussion that includes risks and benefits of the medication, including the potential impact of cannabinoids on the developing brain.
3. Medical cannabinoids should be prescribed as an adjunct to other analgesics and nonpharmacological management strategies, including physical and psychological strategies.
4. Close follow-up is required to evaluate the efficacy and potential adverse drug reaction of the medication and the risk–benefit ratio needs to be regularly reassessed.

This is the largest cohort study yet reported on the use of nabilone in children and adolescents for chronic pain indications. We hope to stimulate discussion of therapeutic options for children with severe pain and for whom current therapies are inadequate, which may lead to increased research in this area.

## Limitations

Limitations of this study include its retrospective design and data collection. The data collection was limited to the documentation available in patients’ medical records. We were unable to exact pain ratings and detailed information about patients’ function from all clinic visits due to incomplete documentation in the outpatient setting. We included subjective data collected from clinicians’ notes, which may be subject to reporting bias. Other limitations include the small number of patients in this study and lack of a control group, which makes it difficult to draw conclusions. The effectiveness of nabilone reported in this study needs to be interpreted with caution, because it was used concurrently with multiple other medications and nonpharmacological treatments that may have contributed to pain control. This study was performed at a single academic tertiary children’s hospital and our results may not be widely generalized to other centers.
